# A Tribute to Professor Katharina Gaus

**DOI:** 10.3389/fbinf.2021.801115

**Published:** 2021-12-15

**Authors:** Marek Cebecauer

**Affiliations:** J. Heyrovsky Institute of Physical Chemistry of the Czech Academy of Sciences, Prague, Czechia

**Keywords:** tribute, single molecule localisation microscopy, Laurdan, cluster analysis, membrane biophysics

*“With new single-molecule tools, and our formidable team, the only limit to what we can achieve is our imagination.”*

It is with great sadness that I report that Professor Dr. Katharina (Kat) Gaus, aged 48, passed away on March 3, 2021. She left with all her energy and enthusiasm, which she constantly devoted to us, her friends, and a broad spectrum of scientific questions. I would like to share with you some brief and personal memories of Katharina Gaus.

I met Kat in Sydney in 2011. She had invited me to stay for 1 month in her lab to learn about single-molecule localization microscopy (SMLM), a modern super-resolution microscopy technique that was already well-established in her laboratory a mere 6 years after appearing in the literature, demonstrating the beauty of biological imaging beyond the diffraction limit. Commercial super-resolution microscopes had just appeared on the market. Her young and productive team was already extensively using SMLM to characterise molecular processes associated with the activation of T lymphocytes (Williamson et al., 2011; Rossy et al., 2013). They were among the very few laboratories that had managed to employ super-resolution microscopy to address key biological questions in such a short time. In fact, this was a typical feature of Kat’s research. She was one of those bold thinkers, who kept bringing new (imaging) technologies into a number of fields, such as immunology, cell biology and virology, to name just a few. To illustrate the impact of Kat’s drive for new technologies, I will mention the two main directions of her research: plasma membrane biophysics and the organisation of signalling molecules on T cells.

Laurdan, a fluorescent membrane probe that is able to sense changes in its environment, was only sparsely used in the community of biophysicists studying synthetic lipid bilayers when Kat harnessed its properties to measure the physical heterogeneity of cellular membranes (Gaus et al., 2003; Gaus et al., 2006). Although the results have been later superseded, Kat and her colleagues continued to improve the Laurdan imaging technology, and the current images certainly are impressive (Ma et al., 2018). Similarly, she pioneered the use of statistical analysis designed for geoinformation studies to characterise surface topography of key players involved in the activation of immune cells (Williamson et al., 2011; Rossy et al., 2013). Cluster analysis used in these early SMLM studies seems a little outdated now and is limited to certain shapes and density levels, but Kat’s team together with her alumni students kept developing more appropriate and advanced cluster analysis methods to achieve more precise information about processes in immune cells (Pageon et al., 2016; Griffié et al., 2017; Hinde et al., 2017; Williamson et al., 2020). Such a continuous effort to improve available technologies underlines Kat’s dedication to advancing the field while delivering excellent science.

Looking back at her publication history and her current team, it is apparent how Kat was able to attract great talent to her laboratory. She built a lab with a mix of biologists, chemists, and physicists at just the right ratio to attack, thanks to this scientific and cultural mixture, important unresolved questions that required unconventional approach(es). This led to several great discoveries and technological improvements, which will serve the community for many years to follow. To highlight contributions to the field of SMLM, it is especially noteworthy how Kat’s team adapted this technique for the quantitative analysis of receptor stoichiometry (Baker et al., 2019), the measurements of intermolecular distances (Coelho et al., 2020), the three-dimensional distribution of molecules (Coelho et al., 2021) and diffusional analysis (Hilzenrat et al., 2020). In collaboration with her partner’s group (Prof. Justin Gooding), they developed a variety of nanostructures for functional and super-resolution imaging and contributed to the application of “click chemistry” in SMLM (Laxman et al., 2021). And I have probably forgotten to refer to several other improvements to this field. However, this long list emphasizes the special position of Kat Gaus in the hearts of microscopists, especially those studying surface molecules on lymphocytes like me. I would like to finish by mentioning that I have never seen Kat frowning. She kept smiling constantly, at least in my presence. I hope that many of you have similar memories. She will be missed, certainly by her collaborators, and the microscopy and SMLM community.

## References

[B1] Baker M. A. B. Nieves D. J. Hilzenrat G. Berengut J. F. Gaus K. Lee L. K. (2019). Stoichiometric Quantification of Spatially Dense Assemblies with qPAINT. Nanoscale 11, 12460–12464. 10.1039/c9nr00472f 31120079

[B2] Coelho S, Baek J, Walsh J, Gooding J. J. Gaus K. (2021). 3D Active Stabilization for Single-Molecule Imaging. Nat. Protoc. 16, 497–515. 10.1038/s41596-020-00426-9 33268882

[B3] Coelho S. Baek J. Graus M. S. Halstead J. M. Nicovich P. R. Feher K. (2020). Ultraprecise Single-Molecule Localization Microscopy Enables *In Situ* Distance Measurements in Intact Cells. Sci. Adv. 6, eaay8271. 10.1126/sciadv.aay8271 32494604 PMC7164934

[B4] Gaus K. Gratton E. Kable E. P. Jones A. S. Gelissen I. Kritharides L. (2003). Visualizing Lipid Structure and Raft Domains in Living Cells with Two-Photon Microscopy. Proc. Natl. Acad. Sci. U S A. 100, 15554–15559. 10.1073/pnas.2534386100 14673117 PMC307606

[B5] Gaus K. Le Lay S. Balasubramanian N. Schwartz M. A. (2006). Integrin-mediated Adhesion Regulates Membrane Order. J. Cel Biol 174, 725–734. 10.1083/jcb.200603034 PMC2064315 16943184

[B6] Griffié J. Shlomovich L. Williamson D. J. Shannon M. Aaron J. Khuon S. (2017). 3D Bayesian Cluster Analysis of Super-resolution Data Reveals LAT Recruitment to the T Cell Synapse. Sci. Rep. 7, 4077. 10.1038/s41598-017-04450-w 28642595 PMC5481387

[B7] Hilzenrat G. Pandžić E. Yang Z. Nieves D. J. Goyette J. Rossy J. (2020). Conformational States Control Lck Switching between Free and Confined Diffusion Modes in T Cells. Biophys. J. 118, 1489–1501. 10.1016/j.bpj.2020.01.041 32097620 PMC7091564

[B8] Hinde E. Thammasiraphop K. Duong H. T. Yeow J. Karagoz B. Boyer C. (2017). Pair Correlation Microscopy Reveals the Role of Nanoparticle Shape in Intracellular Transport and Site of Drug Release. Nat. Nanotechnol 12, 81–89. 10.1038/nnano.2016.160 27618255

[B9] Laxman P. Ansari S. Gaus K. Goyette J. (2021). The Benefits of Unnatural Amino Acid Incorporation as Protein Labels for Single Molecule Localization Microscopy. Front. Chem. 9, 641355. 10.3389/fchem.2021.641355 33842432 PMC8027105

[B10] Ma Y. Benda A. Kwiatek J. Owen D. M. Gaus K. (2018). Time-Resolved Laurdan Fluorescence Reveals Insights into Membrane Viscosity and Hydration Levels. Biophys. J. 115, 1498–1508. 10.1016/j.bpj.2018.08.041 30269886 PMC6257870

[B11] Pageon S. V. Nicovich P. R. Mollazade M. Tabarin T. Gaus K. (2016). Clus-DoC: a Combined Cluster Detection and Colocalization Analysis for Single-Molecule Localization Microscopy Data. Mol. Biol. Cel 27, 3627–3636. 10.1091/mbc.E16-07-0478 PMC5221594 27582387

[B12] Rossy J. Owen D. M. Williamson D. J. Yang Z. Gaus K. (2013). Conformational States of the Kinase Lck Regulate Clustering in Early T Cell Signaling. Nat. Immunol. 14, 82–89. 10.1038/ni.2488 23202272

[B13] Williamson D. J. Burn G. L. Simoncelli S. Griffié J. Peters R. Davis D. M. (2020). Machine Learning for Cluster Analysis of Localization Microscopy Data. Nat. Commun. 11, 1493. 10.1038/s41467-020-15293-x 32198352 PMC7083906

[B14] Williamson D. J. Owen D. M. Rossy J. Magenau A. Wehrmann M. Gooding J. J. (2011). Pre-existing Clusters of the Adaptor Lat Do Not Participate in Early T Cell Signaling Events. Nat. Immunol. 12, 655–662. 10.1038/ni.2049 21642986

